# Advice Taking from Humans and Machines: An fMRI and Effective Connectivity Study

**DOI:** 10.3389/fnhum.2016.00542

**Published:** 2016-11-04

**Authors:** Kimberly Goodyear, Raja Parasuraman, Sergey Chernyak, Poornima Madhavan, Gopikrishna Deshpande, Frank Krueger

**Affiliations:** ^1^Center for Alcohol and Addiction Studies, Department of Behavioral and Social Sciences, Brown University, ProvidenceRI, USA; ^2^Section on Clinical Psychoneuroendocrinology and Neuropsychopharmacology, National Institute on Alcohol Abuse and Alcoholism and National Institute on Drug Abuse, BethesdaMD, USA; ^3^Department of Psychology, George Mason University, FairfaxVA, USA; ^4^Molecular Neuroscience Department, George Mason University, FairfaxVA, USA; ^5^Institute for Defense Analyses, AlexandriaVA, USA; ^6^Auburn University MRI Research Center, Department of Electrical & Computer Engineering, Auburn University, AuburnAL, USA; ^7^Department of Psychology, Auburn University, AuburnAL, USA; ^8^Alabama Advanced Imaging Consortium, Auburn University and University of Alabama, BirminghamAL, USA

**Keywords:** expert advice, functional magnetic resonance imaging (fMRI), effective connectivity, Granger causality, precuneus, posterior insula

## Abstract

With new technological advances, advice can come from different sources such as machines or humans, but how individuals respond to such advice and the neural correlates involved need to be better understood. We combined functional MRI and multivariate Granger causality analysis with an X-ray luggage-screening task to investigate the neural basis and corresponding effective connectivity involved with advice utilization from agents framed as experts. Participants were asked to accept or reject good or bad advice from a human or machine agent with low reliability (high false alarm rate). We showed that unreliable advice decreased performance overall and participants interacting with the human agent had a greater depreciation of advice utilization during bad advice compared to the machine agent. These differences in advice utilization can be perceivably due to reevaluation of expectations arising from association of dispositional credibility for each agent. We demonstrated that differences in advice utilization engaged brain regions that may be associated with evaluation of personal characteristics and traits (precuneus, posterior cingulate cortex, temporoparietal junction) and interoception (posterior insula). We found that the right posterior insula and left precuneus were the drivers of the advice utilization network that were reciprocally connected to each other and also projected to all other regions. Our behavioral and neuroimaging results have significant implications for society because of progressions in technology and increased interactions with machines.

## Introduction

Individuals often encounter situations in their everyday lives when they must rely on advice from others. With new technological advances, advice can come from not only humans, but also automated devices such as a Global Positioning System. For instance, to provide advanced safety measures, the Transportation Safety Administration (TSA) has implemented X-ray luggage scanners and Advanced Imaging Technology (AIT) for screening passengers and exposing potential security threats (Transportation Safety Administration, 2014). Numerous factors can alter the valuation of advice, such as self-confidence (Lee and Moray, 1992; Riley, 1996; Bonaccio and Dalal, 2006), user trust (Rotter, 1967; Mayer et al., 1995; Madhavan and Wiegmann, 2007b), source credibility (i.e., expert) (Birnbaum and Stegner, 1979; Van Swol and Sniezek, 2005; Madhavan and Wiegmann, 2007a) and source reliability/performance (Bonaccio and Dalal, 2006). Additionally, advice errors (false alarms and misses) are variables that can impact decision-making behaviors during advice taking. A false alarm, or an incorrect alert, may be more of a nuisance and not necessarily detrimental, while a miss, or an incorrect alert, may have more serious consequences such as failing to detect an explosive device in a suitcase. For instance, false alarms may cause a “cry wolf effect,” in which individuals may tend to ignore alerts or not respond to them at all (Wickens et al., 2009; Breznitz, 2013), while misses may cause changes in attention allocation strategies (Onnasch et al., 2014). A study comparing false alarms and misses showed that false alarms may hurt overall performance compared to misses (Dixon et al., 2007), providing evidence that there are different perceptions involved with the different error types. Thus the current study only focused on false alarms to not mix the error types. Understanding the effects of error types and how people utilize advice are becoming necessary to provide useful insight for developing safety measures and for appropriate guidelines to predict human behaviors.

Individuals may vary in how they respond to advice and studies have shown that expert advice is more frequently used (Sniezek et al., 2004) and more persuasive than novice advice (Jungermann et al., 2005). In addition, people may respond to advice from automation and humans in similar ways under the premise of a “perfect automation schema,” in which an individual believes that automated aids are near perfect (Dzindolet et al., 2002). Moreover, factors such as dispositional credibility can alter trust between human and machine advisors due to differences in personal traits such as loyalty or benevolence. For example, it has been postulated that association of dispositional credibility is higher for human agents due to evaluation of personal traits, while automated agents may be judged more by performance levels (Madhavan and Wiegmann, 2007a). However, when expectations of reliable advice are altered due to disconfirmation evidence about an advisor’s credibility, decision-making behaviors can be impacted. For example, consistent with disconfirmation theory (Oliver, 1980) decision-making can be affected by initial confirmatory experiences, which can be influenced by bad advice (Staudinger and Buchel, 2013). Furthermore, prior literature on iterative trust games have indicated that trial-and-error learning can modulate choices based on feedback (Delgado et al., 2005) and trust develops over time as reputations are learned and developed (King-Casas et al., 2005). These findings provide evidence that initial beliefs and expectations may be updated over time based upon temporal learning mechanisms.

Despite existing knowledge of the cognitive processes that affect advice taking, the neural mechanisms and the underlying effective connectivity network involved with good and bad advice from human and machine agents framed as experts remains to be explored. Recent neuroimaging studies have investigated the role of expert advice during decision-making (Meshi et al., 2012; Boorman et al., 2013), social learning (Biele et al., 2011; Staudinger and Buchel, 2013) and disobedience (Suen et al., 2014). Furthermore, the neural activity involved with assigning trait and intentions to others (Saxe and Kanwisher, 2003; Mitchell et al., 2006;), self-attributional processes (Cabanis et al., 2013), as well as human-robot interactions during an interactive rock-paper-scissors game (Chaminade et al., 2012) and during observations of social interactions (Wang and Quadflieg, 2015) have been investigated. Overall, key regions associated with the default network (e.g., temporoparietal junction, precuneus, posterior cingulate cortex, medial prefrontal cortex) and the salience network (dorsal anterior cingulate cortex, bilateral insulae) have been identified in playing a role during advice taking, evaluation of personal traits and during human–robot interactions (Krach et al., 2008; Engelmann et al., 2009).

We aimed to elucidate the neural basis of advice utilization from different agents with a between-subjects design and the corresponding effective connectivity in the underlying brain network by combining an X-ray luggage-screening task and functional magnetic resonance imaging (fMRI) with multivariate Granger causality analysis. The focus of this study was to examine the impact of false alarms on advice taking behaviors based on previous evidence that false alarms degrade trust and hurt overall performance more than misses (Dixon et al., 2007). On the behavioral level, we hypothesized that unreliable advice would decrease performance (i.e., accuracy) and advice utilization due to disconfirming evidence about the agents’ perceived expertise. We further assumed that advice utilization would decrease more during bad advice due to disconfirmation evidence stemming from advice-incongruent experiences (i.e., high false alarm rates) (Dixon et al., 2007). This adaption in behaviors would be revealed over time as errors became more apparent due to participants’ reevaluation of the agent’s performance (Skitka et al., 2000). Given the temporal aspect and iterative nature of the task, the inclusion of a time factor allows for an understanding of the influence of time based upon feedback and learning. In addition, we expected that advice utilization would decrease more for the machine agent compared to the human agent due to differences in dispositional credibility between humans and machines (Madhavan and Wiegmann, 2007a). On the neural level, we first predicted activation differences in brain regions associated with attribution of personal traits and dispositions (Harris et al., 2005; Brosch et al., 2013). Secondly, when comparing the human to the machine agent during bad advice over time, we expected regions such as the precuneus and posterior cingulate cortex (Pearson et al., 2011; Cabanis et al., 2013), to be the drivers of the underlying advice utilization network.

## Materials and Methods

### Subjects

Three studies were conducted according to the ethical guidelines and principles of the Declaration of Helsinki. For the normative rating study, twenty-three male students [age (*M* ±*SD*) = 24.0 ± 2.6] from George Mason University (GMU) participated to standardize the X-ray luggage images for the experimental studies. For the behavioral study, 10 volunteers (six males, four females; age = 22.3 ± 2.9) participated to complete an X-ray luggage-screening task without receiving advice. For the fMRI study, 24 healthy right-handed volunteers (13 males, 11 females; age = 20.0 ± 2.6) determined by the Edinburgh Handedness Inventory (Right-handedness: 94.5 ± 7.7) (Oldfield, 1971) participated in the X-ray luggage-screening task and they were randomly assigned to either the human (*n* = 12) or machine (*n* = 12) agent groups for a between-subjects design. All participants gave written consent approved by GMU’s Institutional Review Board and received financial compensation for their participation.

### Stimuli

**FIGURE 1:**
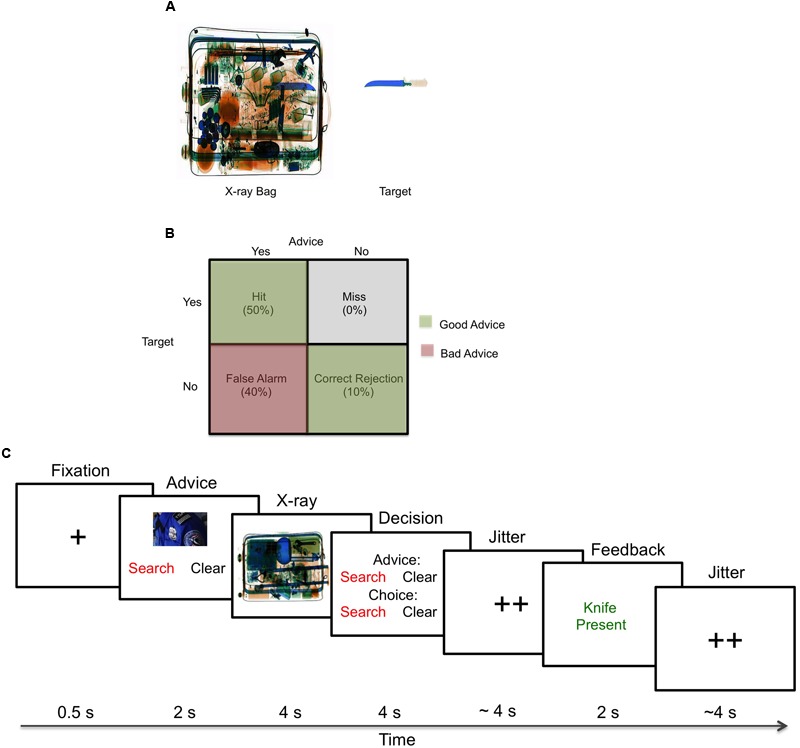
**Experimental setup. (A)** Example stimuli used for the X-ray luggage-screening task. During the normative rating task, participants rated 320 X-ray luggage images (120 target: 60 high clutter, 60 low clutter; 200 non-target: 100 high clutter, 100 low clutter) that contained everyday objects (hair-dryers, clothes, etc.) and a possible target present (five different knives, with one possible per image) based on clutter, difficulty and confidence in finding the knife. **(B)** Decision matrix. Breakdown for each advice type given during the experiment. **(C)** X-ray luggage-screening task. During each trial, participants would first see a fixation cross, advice from one of the agents to “search” or “clear” the bag, an image of the X-ray luggage bag, a decision to accept or reject the advice of the agent to “search” or “clear” the bag, fixation crosses, feedback indicating if their decision was correct or incorrect and lastly, fixation crosses.

During the normative rating study, the participants rated 320 X-ray images based on three dimensions —clutter (4.1 ± 0.3), general difficulty (3.5 ± 0.4), and confidence in finding the target (3.2 ± 0.6)— based on 7-point Likert scales (1 = very low to 7 = very high) (Madhavan and Gonzalez, 2006). From those images, 64 (32 target and 32 non-target) images were chosen for the experimental studies based on the standardized ratings (**Figure 1A**).

### X-ray Luggage-Screening Task

In the X-ray luggage-screening task, participants were asked to search for the presence or absence of a knife. In the behavioral study, participants did not receive advice and performed the task unassisted; participants in the fMRI study received good (advice-congruent) and bad (advice-incongruent) advice from either a human or machine agent. For the fMRI study, the advice was manipulated and the reliability was set to 60% – good advice: 50% hits (correct alerts) and 10% correct rejections (correct non-alerts); bad advice: 40% false alarms (incorrect alerts) (**Figure 1B**).

On each trial, the participants saw a set of phases including a fixation cross (0.5 s), advice from one of the agents to “search” or “clear” the bag (2 s), an image of the X-ray luggage (4 s), a decision to accept or reject the advice of the agent to “search” or “clear” the bag (4 s), jitter (∼4 s), feedback indicating if their decision was correct or incorrect (2.0 s) and lastly, jitter (∼4 s) (**Figure 1C**). The jitter times were generated by an fMRI simulator software^1^ that optimized the timing and consisted of a minimum of 1 s, a maximum of 7 s and an average of 4 s. Participants used response pads to respond and they were given an initial endowment of $40 and each incorrect answer resulted in a deduction of $0.30 from the remaining total. Performance, advice utilization, response times, and monetary deductions were collected during the experiment. The stimuli were presented using E-Prime 2.0 (Psychology Software Tools, Inc^2^.).

### Procedure

#### Pre-experimental Phase

The participants came one to 2 weeks before the fMRI experiment to complete self-report questionnaires as control measures to investigate individual differences between the agent groups. The control measures included: Interpersonal Reactivity Index (IRI, separate facets of empathy) (Davis, 1983), Complacency-Potential Rating Scale (, feelings toward automation) (Singh et al., 1997), National Readiness Technology Scale (NTRS, embracing new technologies) (Parasuraman, 2000), NEO Five-Factor Inventory (NEO-FFI, personality styles) (Costa and McCrae, 1992), and Propensity to Trust (PTT, trust toward automation) (Merritt et al., 2013).

#### Experimental Phase

Before participants completed a practice run for the fMRI experiment, they read descriptions about the human or machine agents (reliability was not disclosed) (Supplementary Table S1). They were then asked to rate their trust in and reliability of the human or machine agent on a 10- point Likert scale (0 = very low, 10 = very high). During the four trials of the practice run, participants familiarized themselves with the X-ray luggage-screening task and the five possible knives that could be present in the bags. The participants then completed two runs (32 trials per run) that were randomized of the experimental task while in the scanner and afterward they were again asked to rate reliability and trust.

#### Post-experimental Session

After the fMRI experiment, participants were asked to rate their confidence in finding the target (i.e., knife) in each of the X-ray luggage images presented during the fMRI experiment on a 10-point Likert scale (1 = very low, 10 = very high).

### fMRI Data Acquisition

Imaging data were acquired on a 3 T head-unit only scanner (Siemens Allegra) with a circularly polarized, transmit/receive head coil at the Krasnow Institute for Advanced Study, GMU, Virginia. The anatomical imaging data were based on a 3D T1 weighted MPRAGE sequence with TR = 2300 ms, TE = 3.37 ms, flip angle = 7°, slice thickness = 1 mm, voxel dimension = 1 mm × 1 mm × 1 mm and number of slices = 160. The functional imaging data were based on a 2D gradient-echo EPI sequence with TR = 2000 ms, TE = 30 ms, flip angle = 70°, slice thickness = 3 mm, voxel dimensions = 3 mm × 3 mm × 3 mm, number of slices = 33 per volume in an axial orientation parallel to the anterior–posterior commissure. The first two volumes were discarded to allow for T1 equilibrium effects and a total of 330 volumes were taken for each run.

### Behavioral Data Analysis

Behavioral data analysis was carried out by Statistical Package for the Social Sciences 20.0 (SPSS 20.0, IBM Corp.) with alpha set to *p* < 0.05 (two-tailed). Data were normally distributed (Kolmogorov–Smirnov test) and assumptions for analyses of variance (Bartlett’s test) were not violated. We first investigated task performance (i.e., accuracy) between the agent groups and the no agent group by employing one-way analysis of variance (ANOVA) with Agent (human, machine, no agent) as the between-subjects factor. Next, we looked at advice utilization, response times and monetary deductions with mixed 2 × 2 × 2 repeated-measures ANOVAs with Advice (good, bad) and Time (run 1, run 2) as within-subjects factors and Agent (human, machine) as the between-subjects factor. In addition, we investigated reliability, trust and confidence ratings with mixed 2 × 2 repeated-measures ANOVAs with Time (pre, post) as the within-subjects factor for the reliability/trust ratings and Target (yes, no) as the within-subjects factor for the confidence ratings and with Agent (human, machine) as the between-subjects factor. Lastly, we performed independent samples *t*-tests between the agent groups to investigate group differences.

### fMRI Data Analysis

The fMRI data analysis was carried out using NeuroElf software^3^ and BrainVoyager QX 2.8 (Brain Innovation). The functional imaging data were preprocessed using Statistical Parametric Mapping 8 (SPM8, Wellcome Department of Cognitive Neurology) functions batched via NeuroElf, including three-dimensional motion correction (six parameters), slice-scan time correction (temporal interpolation) and a mean functional image was computed for each participant across all runs. The mean functional image was then co-registered with the anatomical images using a joint-histogram for the different contrast types. Preprocessing of the anatomical images included segmenting images with a unified segmentation procedure (Ashburner and Friston, 2005) and spatial warping were applied to the functional data to normalize the data to a standard Montreal Neurological Institute (MNI) brain template. Lastly, spatial smoothing (Gaussian filter of 6 mm FWHM) was applied to the images to account for any residual differences across participants. A general linear model (GLM) that was corrected for first-order serial correlations was performed (Friston et al., 2003). The GLM consisted of 36 regressors based on advice utilization (accept, reject) separated by advice (good, bad) and time (run 1, run 2) for each of the five phases (fixation, advice, bag, decision, and feedback) on each trial of the X-ray luggage-screening task and six parametric regressors of no interest for the 3D motion correction (translations in *X, Y, Z* directions, rotations around *X, Y, Z* axes). The regressor time courses were adjusted for the hemodynamic response delay by convolution with a dual-gamma canonical hemodynamic response function (HRF; Buchel et al., 1998). Random-effect analyses were performed at the multi-subject level to explore brain regions associated with the decision and feedback phases.

To reveal brain activations associated with advice utilization, mixed 2 × 2 × 2 ANOVAs on parameter estimates were applied with Advice (good, bad) and Time (run 1, run 2) as within-subjects factors and Agent (human, machine) as the between-subjects factor. For the fMRI results, our main focus was on brain activations during the decision and feedback phases for the three-way interaction since our *a priori* hypotheses was based on the interaction of three factors (advice, time, and agent). However, additional analyses were performed on the main effects for the decision and feedback phases (see Additional Analyses). Activations for the decision and feedback phases were reported after correcting for multiple comparisons using a cluster-level statistical threshold (Cluster-level Statistical Threshold Estimator plugin in BrainVoyager QX), which calculates the minimum cluster size to achieve a false activation probability (α = 0.05) (Forman et al., 1995; Goebel et al., 2006). The voxel-level threshold was set at *p* < 0.005 (uncorrected) and the thresholded map was used for a whole-brain correction criterion based on the estimate of the map’s spatial smoothness and on an iterative procedure (Monte Carlo simulation, 1,000 iterations). The activation clusters were displayed in MNI space on an anatomical brain template reversed left to right.

### Effective Connectivity Analysis

Investigation of the effective (or directional) brain connectivity in the network of activated brain regions was performed through multivariate Granger causality analysis (GCA) using a custom MATLAB^4^ code as previously described by Grant et al. (2014); Kapogiannis et al. (2014) and Lacey et al. (2014). Granger causality is based on a temporal precedence concept (Granger, 1969) that can be applied to multivariate effective connectivity modeling of ROI (region of interest) time courses to predict directional influences among brain regions (Friston et al., 2003; Deshpande et al., 2009; Strenziok et al., 2010; Preusse et al., 2011; Sathian et al., 2011). The model examines the relationship of variables in time, such that given two variables, *a* and *b*, if past values of *a* better predict the present value of *b*, then causality between the variables can be inferred as function of their earlier time points (Roebroeck et al., 2005; Hampstead et al., 2011; Krueger et al., 2011). GCA is advantageous for application of effective connectivity since it is a data-driven approach and there is no requirement for pre-specified connectivity models like dynamic causal modeling (DCM) (Friston et al., 2003; Roebroeck et al., 2005; Deshpande et al., 2009, 2012; Deshpande and Hu, 2012). Recent GCA investigations, including experimental applications (Abler et al., 2006) as well as simulations (Deshpande et al., 2010b; Wen et al., 2013), have shown its advantages and validity for assessing effective connectivity.

Based upon on effective connectivity hypotheses, only those regions that survived the fMRI analysis threshold for the interaction effect Advice (good, bad), Time (run 1, run 2), and Agent (human, machine) for the decision phase were selected as ROIs for the subsequent multivariate GCA. Time series of the BOLD (blood-oxygen-level-dependent) signal for the selected ROIs were extracted around peak activation maxima (sphere of 6 mm × 6 mm × 6 mm), averaged across voxels and normalized across participants, per run. Blind hemodynamic deconvolution of the mean ROI BOLD time series was performed using a Cubature Kalman filter, which has been shown to be extremely efficient for jointly estimating latent neural signals and the spatially variable HRFs (Havlicek et al., 2011). In addition, recent research has shown that this model is not susceptible to over-fitting and produces estimates that are comparable to non-parametric methods (Sreenivasan et al., 2015). Hemodynamic deconvolution removes the inter-subject and inter-regional variability of the HRF (Handwerker et al., 2004) as well as its smoothing effect and therefore, increases the effective temporal resolution of the signal. The resulting latent neural signals were entered into a first order dynamic multivariate autoregressive (dMVAR) model for assessing directed interactions between multiple nodes as a function of time (Wheelock et al., 2014; Grant et al., 2015; Hutcheson et al., 2015) while factoring out influences mediated indirectly in the set of selected ROIs (Stilla et al., 2007; Deshpande et al., 2008, 2010a). A first order model was implemented because of the interest in causal influences arising from neural delays, which are less than a TR (Deshpande et al., 2013). Furthermore, the dMVAR model’s coefficients were allowed to vary as a function of time. Therefore, directional connectivity between selected ROIs can be estimated using the dMVAR model coefficients at each specific time instant. Since the experimental design consists of conditions of interest as well as inter-trial rest intervals, condition-specific connectivity values can be obtained as sample distributions by aggregating model coefficients corresponding to all time instants for specific conditions (Sathian et al., 2013; Grant et al., 2014, 2015; Lacey et al., 2014; Wheelock et al., 2014; Hutcheson et al., 2015; Feng et al., 2016). Granger connectivity (GC) path weights, i.e., the model coefficients, for conditions of interest (bad advice) for each agent (human, machine) were extracted.

**FIGURE 2:**
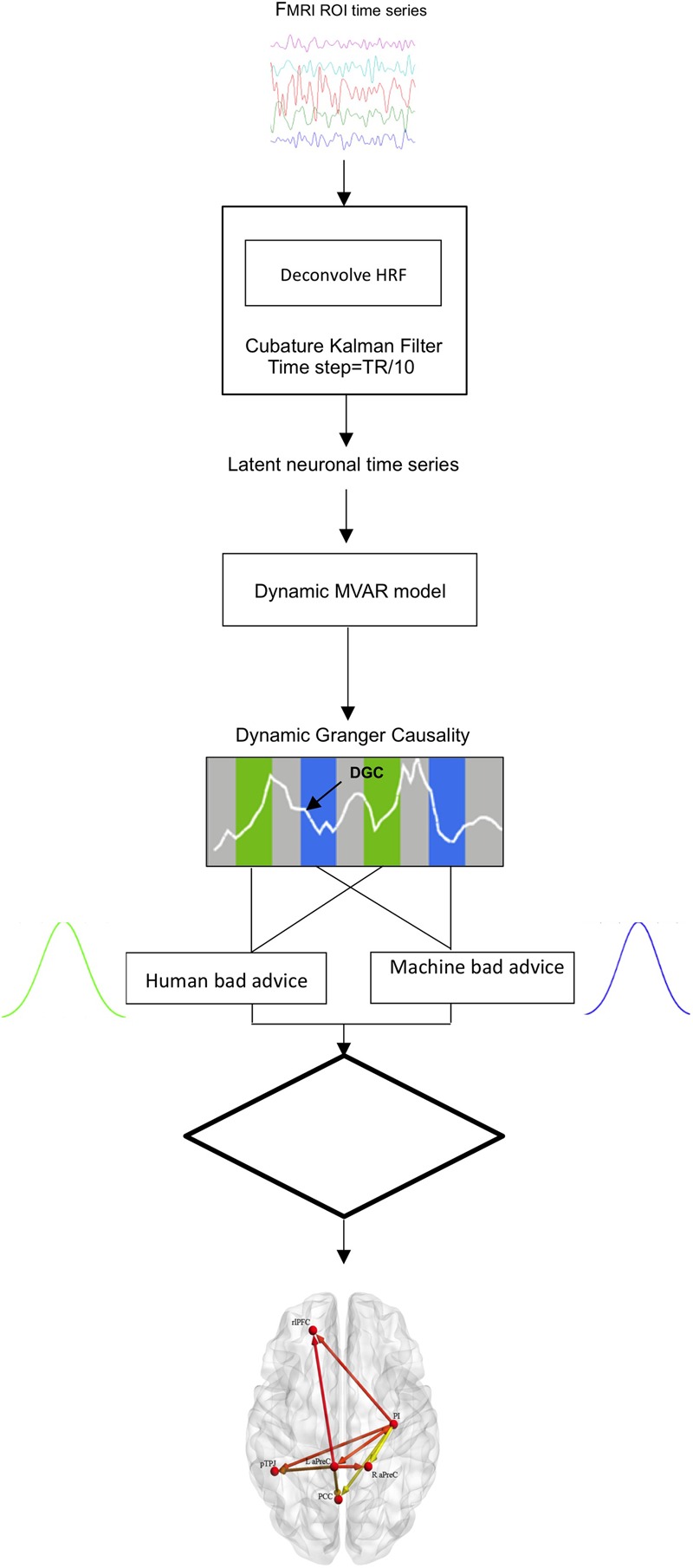
**Schematic illustrating the effective connectivity analysis pipeline**.

Those corresponding GC path weights were populated into two samples and independent samples *t*-tests were employed to reveal the condition-specific modulations of connectivity [*q*(FDR) < 0.05) (Benjamini and Hochberg, 1995), i.e., those paths which had significantly different effective connectivity between human and machine agents while receiving bad advice (**Figure 2**). Since GCA is a data-driven approach, the condition-specific modulation was specifically chosen for analysis based upon our fMRI results. Effective connectivity of brain regions (i.e., nodes, edges) was displayed on a brain surface using BrainNet Viewer (www.nitrc.org/projects/bnv/), a graphical interface visualization tool (Xia et al., 2013). Lastly, we performed bivariate Spearman’s correlations to identify associations between behavioral measures (i.e., advice utilization) and GC path weights for the human- and machine-agent groups.

## Results

### Behavioral Results

**FIGURE 3:**
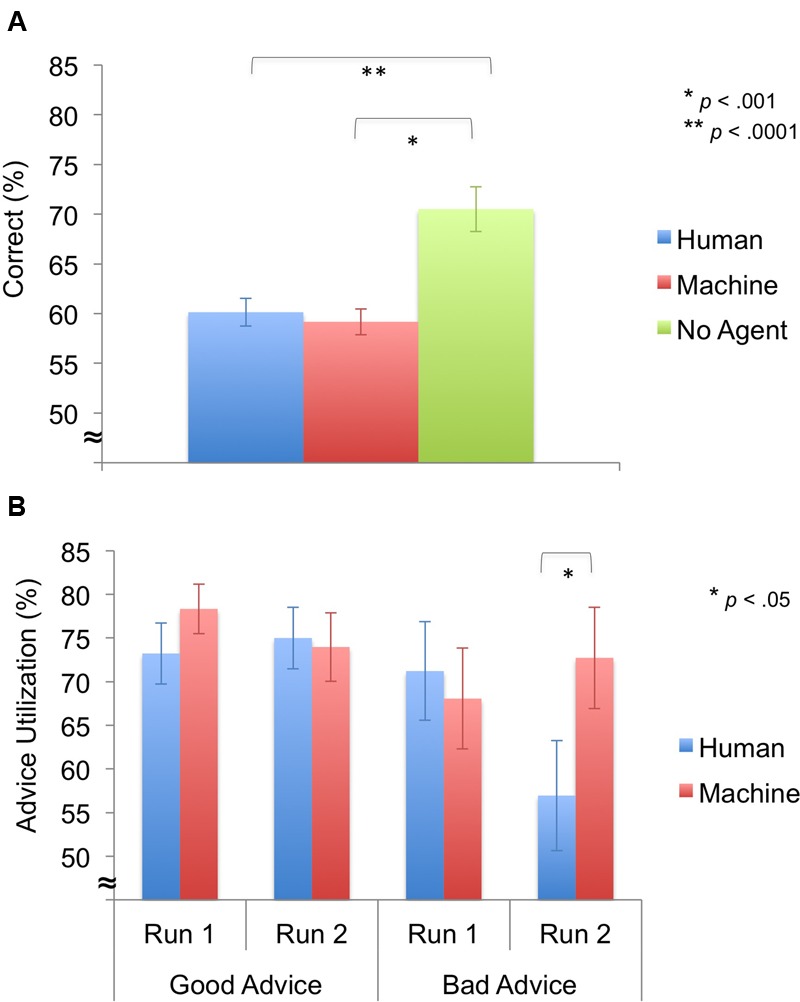
**Behavioral results for decision phase (*M* ± *SEM*). (A)** Task performance. The no agent group performed better than human- and machine-agent groups. **(B)** Advice utilization. Advice utilization during bad advice from the human agent was significantly lower during run 2 compared to the machine agent.

First, we compared the performance between the agent groups and the no advice group by employing a one-way ANOVA with Agent (human, machine, no agent) as between-subjects factors. A significant main effect of Agent [*F*(2,31) = 13.85, *p* < 0.0001] was revealed, and *post hoc* testing revealed that the no agent group performed better than the human-agent group [*t*(20) = -4.06, *p* = 0.001] and the machine-agent group [*t*(20) = -4.54, *p* < 0.0001]. (**Figure 3A**).

Next, we looked at advice utilization, response times, and monetary deductions For *advice utilization*, a significant main effect of Advice was revealed [*F*(1,22) = 7.63, *p* = 0.011], indicating that participants accepted good advice more than bad advice. In addition, a significant three-way interaction of Advice × Time × Agent was identified [*F*(1,22) = 5.06, *p* = 0.035], but no significant main effects of Agent [*F*(1,22) = 0.65, *p* = 0.429] or Time [*F*(1,22) = 2.30, *p* = 0.144] and no significant two-way interaction effects of Advice × Agent [*F*(1,22) = 0.56, *p* = 0.463], Time × Agent [*F*(1,22) = 2.54, *p* = 0.125], and Advice × Time [*F*(1,22) = 0.40, *p* = 0.536] (**Figure 3B**) were found. Follow-up 2 × 2 ANOVAs showed a significant interaction effect of Time × Agent for bad advice [*F*(1,22) = 5.63, *p* = 0.027], but not for good advice [*F*(1,22) = 1.23, *p* = 0.279]. Follow-up independent samples *t*-tests revealed that the human-agent group accepted bad advice less than the machine-agent group during run 2 [*t*(22) = -1.84, *p* = 0.040].

**FIGURE 4:**
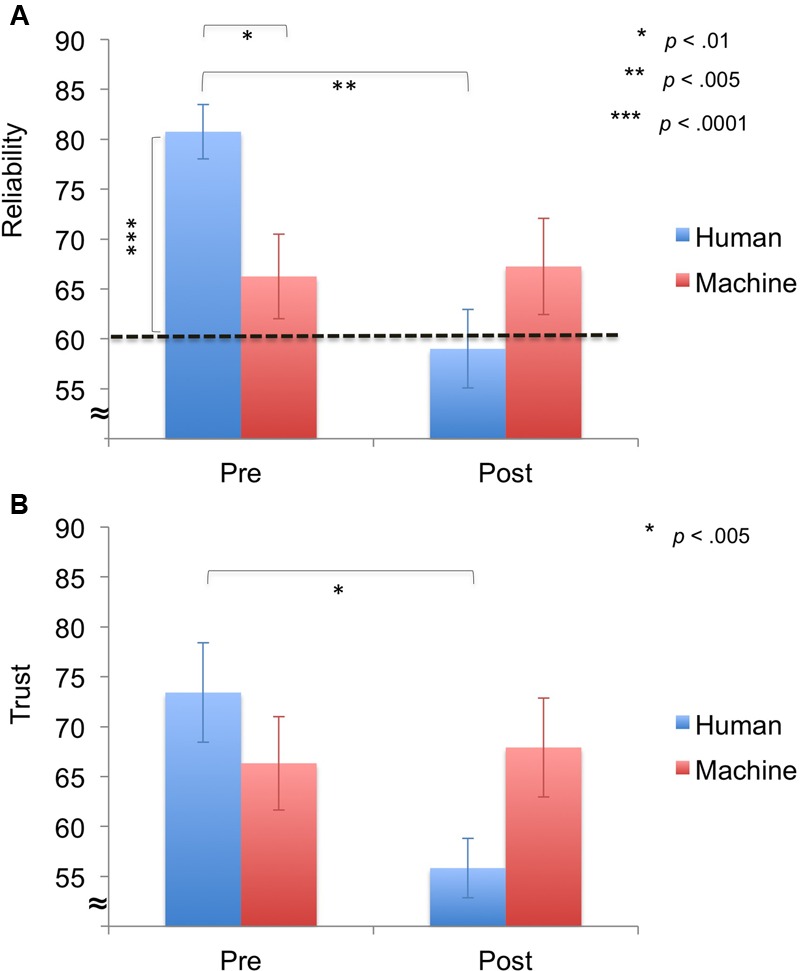
**Results for ratings (*M* ± *SEM*). (A)** Pre- and Post-reliability. Pre-reliability was higher for the human agent compared to the machine agent. For the human agent, perceived pre-reliability was significantly higher than the actually reliability of the agent (60%) and post-reliability ratings significantly decreased. **(B)** Pre- and post-trust. Post-trust was significantly lower than pre-trust for the human agent.

In addition, we looked at pre- and post-experiment ratings (reliability, trust) using repeated-measures ANOVAs with Time (run 1, run 2) and Agent (human, machine) as factors. The *reliability ratings* showed no significant main effect of Agent [*F*(1,22) = 0.62, *p* = 0.439], but a significant main effect of Time [*F*(1,22) = 6.54, *p* = 0.018] and a significant interaction effect of Time × Agent [*F*(1,22) = 7.86, *p* = 0.010] (**Figure 4A**). *Post hoc* testing revealed that the human agent’s pre-reliability was rated higher than the machine’s pre-reliability [*t*(22) = 2.87, *p* = 0.009] and the human’s reliability ratings decreased from pre- to post-experiment [*t*(11) = 4.10, *p* = 0.002]. Furthermore, one-sample *t*-tests on perceived versus actual reliability (60%) of the agent showed that pre-reliability ratings were significantly higher than the actual reliability for the human agent [*t*(11) = 6.79 *p* < 0.0001].

For *trust ratings*, no significant main effects of Agent [*F*(1,22) = 0.26, *p* = 0.615] and Time [*F*(1,22) = 3.96, *p* = 0.059] were observed, but a significant interaction effect of Time × Agent [*F*(1,22) = 5.89, *p* = 0.026] was demonstrated (**Figure 4B**). *Post hoc* testing revealed that trust ratings significantly decreased from pre- to post-experiment for the human agent [*t*(11) = 4.18, *p* = 0.002].

### Neuroimaging Results

**FIGURE 5:**
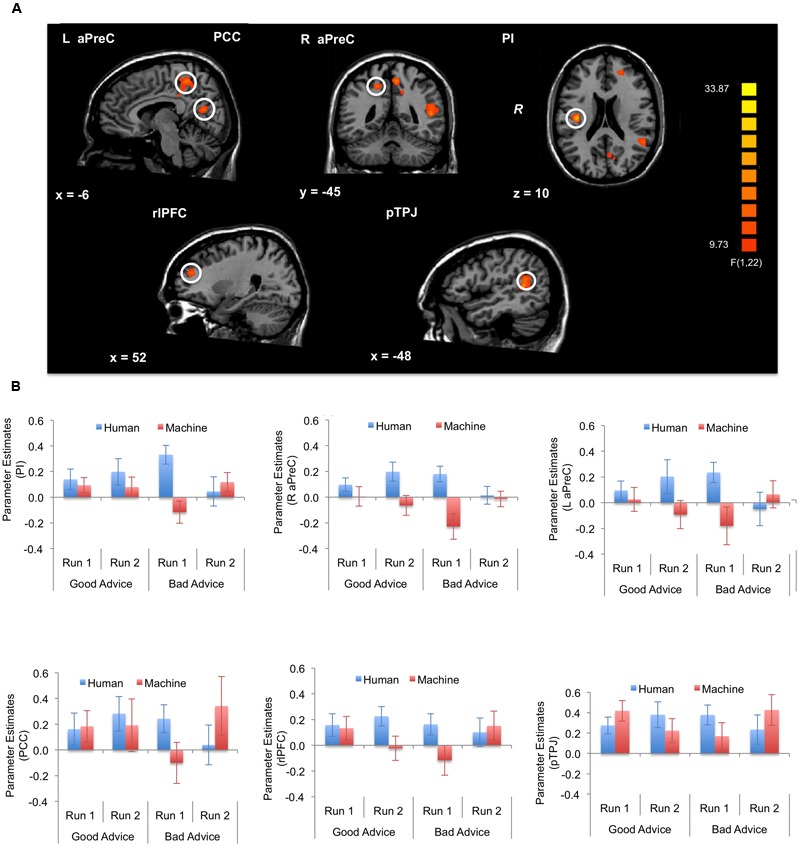
**Brain activations during decision phase (α < 0.05, *k* = 21).** The three-way interaction (Advice × Run × Agent) during the decision phase significantly activated the right posterior insula (PI), right anterior precuneus (aPreC), left aPreC, left posterior cingulate cortex (PCC), left rostrolateral prefrontal cortex (rlPFC), and left posterior temporoparietal junction (pTPJ). The activation pattern indicates higher activation for the human- compared to machine-agent group for bad advice during run 1. The bar plots shown are for visualization purposes.

**FIGURE 6:**
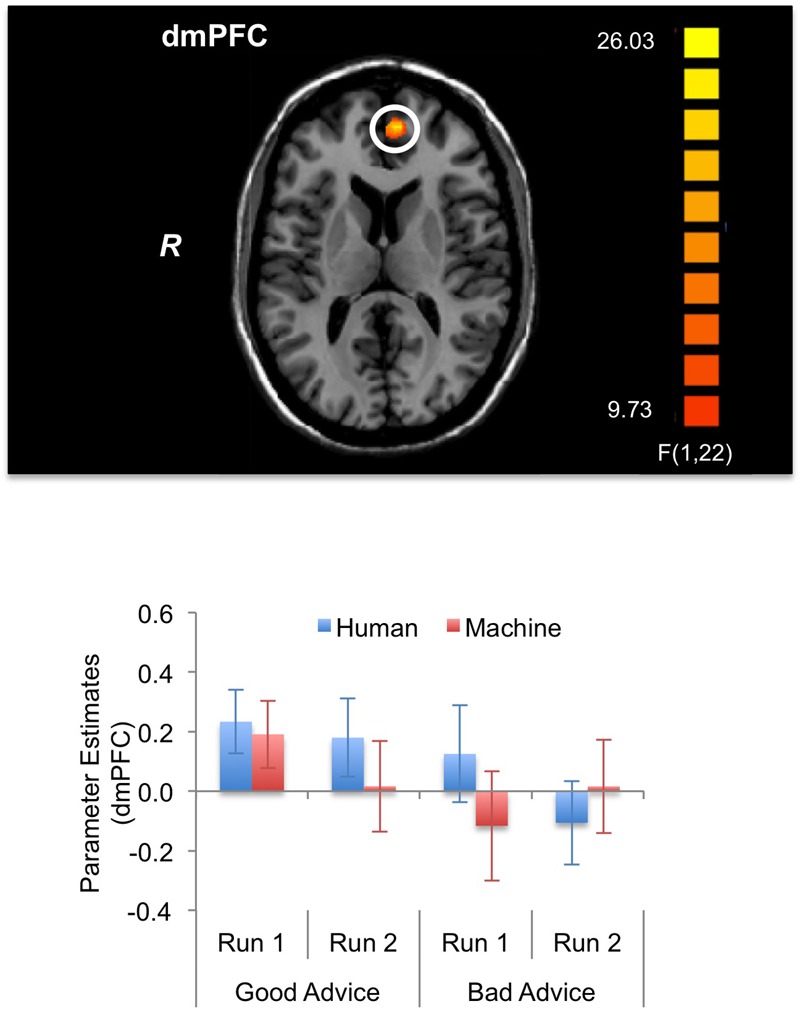
**Brain activations during feedback phase (α < 0.05, *k* = 14).** The three-way interaction (Advice × Run × Agent) during the feedback phase significantly activated the left dorsomedial prefrontal cortex (dmPFC). The activation pattern shows lower activation for bad advice compared to good advice during run 2 for the human agent. The bar plots shown are for visualization purposes.

For the fMRI results, we looked at brain activations during the decision and feedback phases for the three-way interaction. For the *decision phase*, a significant three-way interaction effect (α < 0.05, *k* = 21) was found in the right (R) posterior insula (PI) (BA 13); R anterior precuneus (aPreC) (BA 5/7), left (L) aPreC (BA 5/7); L posterior cingulate cortex (PCC) (BA 30/31); L rostrolateral prefrontal cortex (rlPFC) (superior frontal gyrus: SFG; BA 10); and L posterior temporoparietal junction (pTPJ) (superior temporal gyrus: STG; BA 22) (**Figure 5**; **Table 1**). The results indicate that there was higher activation during run 1 for the human-agent group compared to machine-agent group during bad advice. For the *feedback phase*, a significant three-way interaction (α < 0.05, *k* = 14) was found in the L dorsomedial prefrontal cortex (dmPFC) (medial frontal gyrus: MFG; BA 9/10) showing higher activation for the human agent during run 2 for good compared to bad advice (**Figure 6**; **Table 1**). Note that no further *post hoc* comparisons were performed on the extracted data from the decision or feedback phases to avoid non-independent analyses, or double dipping (Kriegeskorte et al., 2009).

**Table 1:** Brain regions associated with the three-way interaction.

	*F*(1,22) value	Cluster size (mm^3^)	*x*	*y*	*z*
**Decision phase**					
*(Advice × Run × Agent)*					
Right posterior insula	32.86	854	36	-15	21
Right anterior precuneus	18.65	593	18	-42	45
Left anterior precuneus	21.52	2214	-6	-42	51
Left posterior cingulate cortex	24.96	607	-3	-63	15
Left rostrolateral prefrontal cortex	17.34	692	-21	45	21
Left posterior temporoparietal junction	23.58	1678	-48	-45	9
**Feedback phase**					
*(Advice × Run × Agent)*					
Left dorsomedial prefrontal cortex	25.03	655	-6	51	12

*Brain regions showing significant activation clusters associated during the decision (minimum cluster of 21) and feedback (minimum cluster of 14) phases (α < 0.05, cluster-level threshold corrected). PI, posterior insula (BA 13); aPreC, anterior precuneus (BA 5/7); PCC, posterior cingulate cortex (BA 30/31); rlPFC, rostrolateral prefrontal cortex (BA 10); pTPJ, posterior temporoparietal junction (BA 22); dmPFC, dorsomedial prefrontal cortex (BA 9/10).*

### Effective Connectivity Results

**FIGURE 7:**
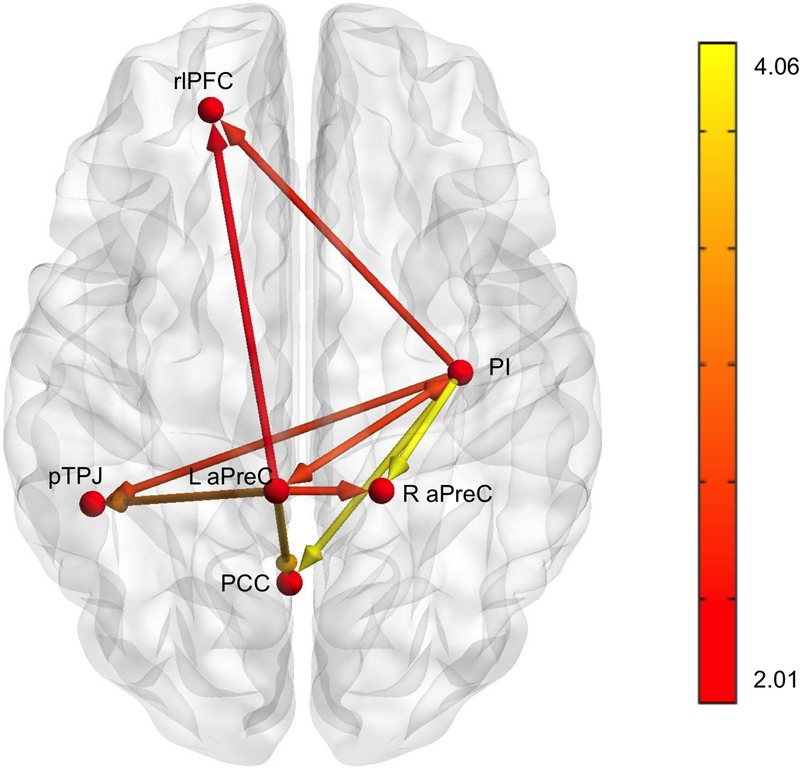
**Results for multivariate Granger causality analysis.** The effective connectivity network for bad advice during the decision phase for run 1 when comparing the human with machine agent showed that the PI (posterior insula) and L aPreC (anterior precuneus) were drivers of the network and also the source ROIs for all other target ROIs (R aPreC, PCC posterior cingulate cortex), rlPFC (rostrolateral prefontal cortex), and pTPJ (posterior temporoparietal junction). Note that all connections survived *q*(FDR) < 0.05, except the connections to rlPFC that survived *q*(FDR) < 0.08. The color bar represents the *t*-value of the comparisons shown in **Table 2**.

Based on our fMRI results, we implemented multivariate GCA to identify effective connectivity among brain regions during the decision phase when comparing the human with the machine agent during bad advice for run 1 [all connections survived *q*(FDR) < 0.05, except the connections to the L rlPFC that survived *q*(FDR) < 0.08] (**Table 2**). Analysis for the feedback phase was not done due to the fact that only one region survived for the fMRI results. The L aPreC and PI were identified as the source ROIs; they were the drivers of the network making reciprocal connections to each other, while also both sending output connections to all target ROIs (R aPreC, PCC, rlPFC, and pTPJ) (**Figure 7**). The correlation analysis for advice utilization and GC path weights for both groups revealed no significant results.

**Table 2:** Path weights for Granger causality Analysis.

		Path weight	
Source	Target	Human	Machine	*t-*value	*p-*value
PI	R aPreC	0.23	0.18	4.06	2.80 × 10^-5^
	L aPreC	0.18	0.19	2.57	5.16 × 10^-3^
	PCC	0.27	0.18	3.96	4.16 × 10^-5^
	rlPFC	0.16	0.18	2.32	1.04 × 10^-2^
	pTPJ	0.17	0.15	2.52	6.02 × 10^-3^
L aPreC	PI	0.18	-0.17	2.42	7.80 × 10^-3^
	R aPreC	0.18	-0.12	2.44	7.51 × 10^-3^
	PCC	0.20	-0.15	3.47	2.79 × 10^-4^
	rlPFC	0.16	-0.15	2.01	2.22 × 10^-2^
	pTPJ	0.24	-0.21	3.12	9.39 × 10^-4^

*The path weights displayed show significant effective connectivity paths that are stronger in the human-agent group compared to the machine-agent group during run 1 [all connections survived *q*(FDR) < 0.05, except the connection to rlPFC that survived *q*(FDR) < 0.08]. The directionality of the connectivity is shown in the first two columns, with the source column showing the ROIs that predict activation in the target column ROIs. The strength of connectivity is given by the mean path weights in the third column. PI, posterior insula; aPreC, anterior precuneus; PCC, posterior cingulate cortex; rlPFC, rostrolateral prefrontal cortex; pTPJ, posterior temporoparietal junction.*

### Additional Analyses

#### Behavioral Results

For *response times*, significant main effects of Advice [*F*(1,22) = 12.26, *p* = 0.002] and Time [*F*(1,22) = 5.85, *p* = 0.024] were found, indicating that responses were faster during good compared to bad advice and during run 2 compared to run 1 (Supplementary Figure S1a). A marginally significant interaction effect was found for the interaction of Time × Agent [*F*(1,22) = 4.35, *p* = 0.049], but no significant main effect of Agent [*F*(1,22) = 0.49, *p* = 0.491] and no significant interaction effects of Advice × Agent [*F*(1,22) = 0.10, *p* = 0.758], Advice × Time [*F*(1,22) = 0.07, *p* = 0.798], and Advice × Time × Agent [*F*(1,22) = 0.06, *p* = 0.811] were found.

For *monetary deductions*, a significant main effect of Advice [*F*(1,22) = 292.45, *p* < 0.0001] was revealed, indicating that deductions were higher during bad advice compared to good advice (Supplementary Figure S1b). In addition, a marginally significant interaction effect of Time × Agent was found [*F*(1,22) = 4.61, *p* = 0.043], but no significant main effects of Time [*F*(1,22) = 0.31, *p* = 0.583] and Agent [*F*(1,22) = 1.56, *p* = 0.224], or interaction effects of Advice × Agent [*F*(1,22) = 0.10, *p* = 0.758], Advice × Time [*F*(1,22) = 0.10, *p* = 0.921], and Advice × Time × Agent [*F*(1,22) = 0.09, *p* = 0.768] were found.

For *confidence ratings*, no main effect of Agent [*F*(1,22) = 4.16, *p* = 0.054] or significant interaction effect of Target × Agent [*F*(1,22) = 2.46, *p* = 0.131] were found, but a significant main effect of Target [*F*(1,22) = 53.44, *p* < 0.0001] was revealed, indicating that confidence was rated higher on target bags compared to non-target bags (Supplementary Figure S2).

Finally, we analyzed at differences in control measures (e.g., demographic measures and questionnaires) with independent samples *t*-tests. No significant group differences were identified for any of the control measures (Supplementary Table S2).

#### Neuroimaging Results

Further contrasts were performed for advice (good, bad), decision (accept, reject) and performance (correct, incorrect). For the main effect of advice during the *decision phase*, a significant cluster (α < 0.05, *k* = 21) was revealed in the right orbitofrontal cortex (superior frontal gyrus, BA 11). For the *feedback phase*, significant activation clusters for the main effect of advice were found in right middle frontal gyrus (BA 6/8), right superior parietal lobule (BA 7), right putamen, right posterior cingulate cortex (BA 30), right head of the caudate, left orbitofrontal cortex (medial frontal gyrus, BA 11), left precentral gyrus (BA 4), left subcallosal gyrus (BA 34), left middle frontal gyrus (BA 6), left dorsolateral prefrontal cortex (middle frontal gyrus, BA 46) and left inferior frontal gyrus (BA 47) (Supplementary Table S3).

For decision (accept > reject) during the *decision phase* (α < 0.05, *k* = 34), significant activation clusters were found in the right superior temporal gyrus (BA 41), right inferior parietal lobule (BA 40), right postcentral gyrus (BA 3), right precentral gyrus (BA 4), right lingual gyrus (BA 18), left cingulate gyrus (BA 24), left postcentral gyrus (BA 5), left superior temporal gyrus (BA 22) (Supplementary Table S4).

For performance (correct > incorrect) during the *feedback phase* (α < 0.05, *k* = 57), significant activation clusters were shown in the right inferior parietal lobule (BA 40), right frontal eye fields (middle frontal gyrus, BA 8), right middle occipital gyrus (BA 18), right putamen, right cingulate gyrus (BA 31), left frontal eye fields (BA 8), left dorsolateral prefrontal cortex (middle frontal gyrus, BA 46), left inferior occipital gyrus (BA 18), left angular gyrus (BA 39) (Supplementary Table S5).

## Discussion

The purpose of this research was to understand the neural basis and corresponding effective connectivity network involved during advice utilization from human and machine agents framed as experts. To provide a greater understanding of the behavioral and neural underpinnings associated with advice taking, we manipulated agent reliability with a high false alarm rate to reveal the decision-making processes during good and bad advice. We first revealed that unreliable advice decreased performance, which has been previously reported by other behavioral studies investigating advice differences between humans and machines (Dzindolet et al., 2002; Madhavan and Wiegmann, 2007a). An earlier study investigating credibility found that advice utilization decreased for expert automation but not for expert humans; however, this study focused entirely on misses and false alarms, which could account for any differences between these earlier findings and ours (Madhavan and Wiegmann, 2007a). In addition, a study investigating perception during a contrast-detection task showed that false alarms evoked more cortical activity when compared to misses, which supports the notion that participants’ percepts may vary when presented with different types of errors (Ress and Heeger, 2003). In our study, we focused only on false alarms since there is evidence of distinct neuronal activity associated with false alarms when compared to misses and behavioral studies have demonstrated differences between the two error types (Dixon et al., 2007; McBride et al., 2014).

Contradictory to our hypothesis, the behavioral results revealed that the decline in advice utilization was greater for the human agent compared to the machine agent. We expected that advice utilization would degrade faster for the machine agent because of differences in association of dispositional credibility; however, our results indicate that false alarms weighed more heavily on the human-agent group. Our findings provide evidence that although assignment of personal traits may have been higher for the human agent, the prevalence of false alarms may have altered evaluations of performance levels due to the type of error presented. Furthermore, to reveal any preconceived notions that participants had about the human and machine agents, we examined whether the perceived pre-reliability differed from the actual reliability for each agent. Interestingly, the human agent’s pre-reliability was rated significantly higher than the actual reliability, showing that the human-agent group expected their advisor to be more reliable. Our finding supports other behavioral studies that indicate that preconceived notions can influence participants’ perceptions of advice (Madhavan and Wiegmann, 2007b). Furthermore, these findings indicate that participants interacting with the human agent could have perceivably built a mental model of their expectations about the agent’s credibility and deviations from expected behavior likely caused a reevaluation of the human agent’s performance (Burgoon, 1993). The change in perspectives would ultimately cause a shift toward self-reliance and possibly increased responsibility/accountability for the outcome of their decisions (Dzindolet et al., 2002). Post-reliability ratings for the human-agent group showed a shift toward the actual reliability of the agent, which indicates that the human-agent group was able to discern the agent’s performance and recalibrate their expectations. Moreover, post-trust was lower than pre-trust for human agent, supporting previous evidence that false alarms degrade trust (Dixon et al., 2007; Rice and McCarley, 2011). Lastly, our results cannot be explained by any of our control measures or confidence ratings because we found no differences between the agent groups.

Moreover, our results revealed that advice utilization decreased during bad advice compared to good advice. Since bad advice was advice-incongruent, it could have created a mismatch between what the participants perceived and what they were advised, resulting in disconfirmation experiences. The discrepancies during advice-disconfirmation experiences most likely lead to skepticism during bad advice and ultimately degradation of advice utilization. As a consequence, response times for both groups were slower during bad advice, since participants had more conflicting perceptual processes (advice-incongruencies). In addition, monetary deductions were higher overall for bad advice, indicating that bad advice caused participants to make more erroneous decisions.

Subsequently, we identified the neural basis and effective connectivity of the underlying brain network associated with advice utilization. On the neural level, we had two expectations regarding brain activity. First, we expected activation differences in regions associated with attribution of personal traits and dispositions, (Harris et al., 2005; Brosch et al., 2013), and secondly, when comparing the agent groups during bad advice over time, brain regions such as the precuneus and posterior cingulate cortex would be the drivers of the advice utilization network. Our neuroimaging results revealed brain regions associated with domain-general large-scale networks, such as the default-mode network (left pTPJ, bilateral aPreC, left PCC) typically engaged in social evaluations, the salience network (AI) with the PI interaction for detection of internal and external salient events, and the central-executive network (left rlPFC) implicated in higher-order executive functions (Menon, 2011). Similarly to our fMRI hypotheses, on the effective connectivity level, we theorized that a network to be differentially involved when comparing the human to the machine agent for bad advice during run 1. Our effective connectivity analysis revealed that left aPreC and PI were drivers of the network that were reciprocally connected to each other. The aPreC and PI acted as centralized hubs of the network, presumably by integrating social evaluations (e.g., judgments about other’s intentions and personal traits) (Cavanna and Trimble, 2006) with interoception (e.g., recruitment of physiological responses to environmental cues) (Kurth et al., 2010). Previous evidence supports the notion that integration of subjective mental states (PreC) and information about internal bodily states (anterior insula, AI) are important for awareness of one’s emotional state (Terasawa et al., 2013). Since participants interacting with the human agent could have had greater conceptualization of the discrepancies between the actual and perceived reliability, this could have led to evaluations about accepting or rejecting the unreliable advice due to interoception (PI) (Engelmann et al., 2009; Menon and Uddin, 2010; Xue et al., 2010; Kelly et al., 2012) in conjunction with association of personal traits (aPreC) during interactions with the agent.

Furthermore, our effective connectivity results indicated that both hubs (left aPreC, PI) had directional influences on all other regions (right aPreC, left pTPJ, PCC, and left rlPFC) to guide decision-making processes during advice utilization. PreC activation has been identified during a comparison of other- vs. self-attribution, showing the involvement of this region during causal attributions toward another (Farrer and Frith, 2002). In addition, PCC activation has been implicated in adapting behaviors (Pearson et al., 2011) and self-reflection (Johnson et al., 2002), while the pTPJ has been shown to be activated during social cognitions such as determining intentionality of others (Mars et al., 2012). Furthermore, we found directional influences to the rlPFC, which is part of the central-executive network and has shown to be involved in reasoning (Christoff et al., 2001) and while making uncertain decisions (Badre et al., 2012).

During advice taking, individuals may decide to discount or utilize the advice given to them and this can vary depending on different factors such as the source or type of advice. For instance, studies investigating tracking of expertise for humans and algorithms (Boorman et al., 2013) and perceptions of competence during advice evaluations (Schilbach et al., 2013) found areas associated with the mentalizing network and salience networks (e.g., ACC, precuneus). Our fMRI results provide a greater discernment of the distinct mental processes involved during advice acceptance from different sources of advice and the behavioral changes that occurred with each agent (e.g., less degradation of advice utilization with the machine agent). One way in which fMRI can inform us about cognitive processes is by allowing us to compare two tasks to determine if they engage similar or distinct mental processes (Mather et al., 2013). Our findings showed that behaviorally, participants responded differently to each agent and similarly, our fMRI findings also demonstrated that distinct mental mechanisms were involved during advice taking with each agent. The fact that there was coinciding brain activity along with behavioral responses for both the human and machine agent points to different mechanistic processes when participants utilize advice from different sources. Furthermore, other fMRI studies investigating expert advice have shown activation in PCC and PreC during no advice conditions (Engelmann et al., 2009) and in regions such as PCC, insula and medial frontal gyrus when comparing advice vs. no advice in experts and peers (Suen et al., 2014); however, we did not expect equivalent results since our experimental design looked at differences between humans and machines.

In addition to our results for the decision phase, we also expected participants to have a heightened awareness of bad advice due to feedback, which would ultimately lead to a behavioral adjustment in advice utilization over time. During the feedback phase, we found activation in the dmPFC, which coincides with another study that showed dmPFC activity during feedback after iterative trials with the same advisor (Behrens et al., 2008). The dmPFC has been shown to be involved with social cognition (Amodio and Frith, 2006) and during inferences about other’s goals and traits (Krueger et al., 2008; Van Overwalle, 2009). In our study, participants interacting with the human agent showed lower dmPFC activation during bad compared to good advice toward the end of the experiment, which shows that, as participants ascertained that the human agent was unreliable, they could have placed lower value on bad advice while receiving feedback.

Our study had a few limitations that should be addressed. First, we looked at differences between good and bad advice by manipulating agent reliability with only false alarms. Future studies could elaborate on our findings by investigating how misses degrade advice utilization between humans and machines and the effective connectivity network associated with those differences. Furthermore, to prevent cognitive anchoring, or the tendency to rely too heavily on the first piece of information acquired, we had participants receive advice before they made their decisions, rather than receiving advice after they made their decisions. Cognitive anchoring has been shown to decrease reliance on automated aids during self-generated decisions (Madhavan and Wiegmann, 2005) and future studies could investigate this phenomena by implementing a paradigm where participants receive advice after they make their decisions. Additionally, our sample size was on the lower side (given that we had a between-subjects design) and future studies could include more participants. Increasing the sample size would allow future studies to incorporate an analysis like a Bayesian learning model to provide greater insight into participant’s learning patterns. Moreover, in our study, learning occurred rapidly due to feedback and thus a learning model was not feasible for our analysis. Future study paradigms could directly compare learning models to assess any differences in adaptive behaviors. Lastly, our voxel-level threshold was set at *p* < 0.005, and while recent evidence has shown that false positive rates may be particularly high at that threshold (Eklund et al., 2016), future studies need to assess other fMRI software packages (e.g., BrainVoyager) to see if the same question of validity arises.

In summary, our findings provide extensive insight into underlying factors involved with advice utilization from humans and machines and the differences that account for those behaviors. Our results have significant implications for society because of progressions in technology and increased interactions with machines. A greater discernment of the various facets involved with machine interactions will ultimately serve to calibrate behavioral responses and to optimize future safety guidelines. For instance, there could be training protocols designed for both individuals giving and receiving advice. Advisors could be trained to have high search rates for bags (higher false alarms/lower misses), while advisees could be trained to have greater vigilance (less reliance) to minimize complacent behaviors, which can result in higher miss rates. By providing the appropriate training, this can help to improve security measures and ultimately prevent potential catastrophic disasters.

## Author Contributions

KG and SC acquired the data for analysis. KG, RP, and FK contributed to the conception of the design. KG, RP, SC, PM, GD, and FK contributed to interpretation of the data. KG, RP, SC, PM, GD, and FK contributed to drafting of the work and revising it critically. KG, RP, SC, PM, GD, and FK approved the final version to be published. KG, RP, SC, PM, GD, and FK agreed to be accountable for all aspects of the work.

## Conflict of Interest Statement

The authors declare that the research was conducted in the absence of any commercial or financial relationships that could be construed as a potential conflict of interest.

## Footnotes

1 http://www.mccauslandcenter.sc.edu/crnl/tools/fmristim

2 http://www.pstnet.com/eprime.cfm

3 http://neuroelf.net

4 www.mathworks.com
