# Supplementary figures and images for: Advice Taking from Humans and Machines: An fMRI and Effective Connectivity Study
Source: Front Hum Neurosci. 2016 Nov 4;10:542. doi: 10.3389/fnhum.2016.00542 (PMC5095979; doi:10.3389/fnhum.2016.00542)

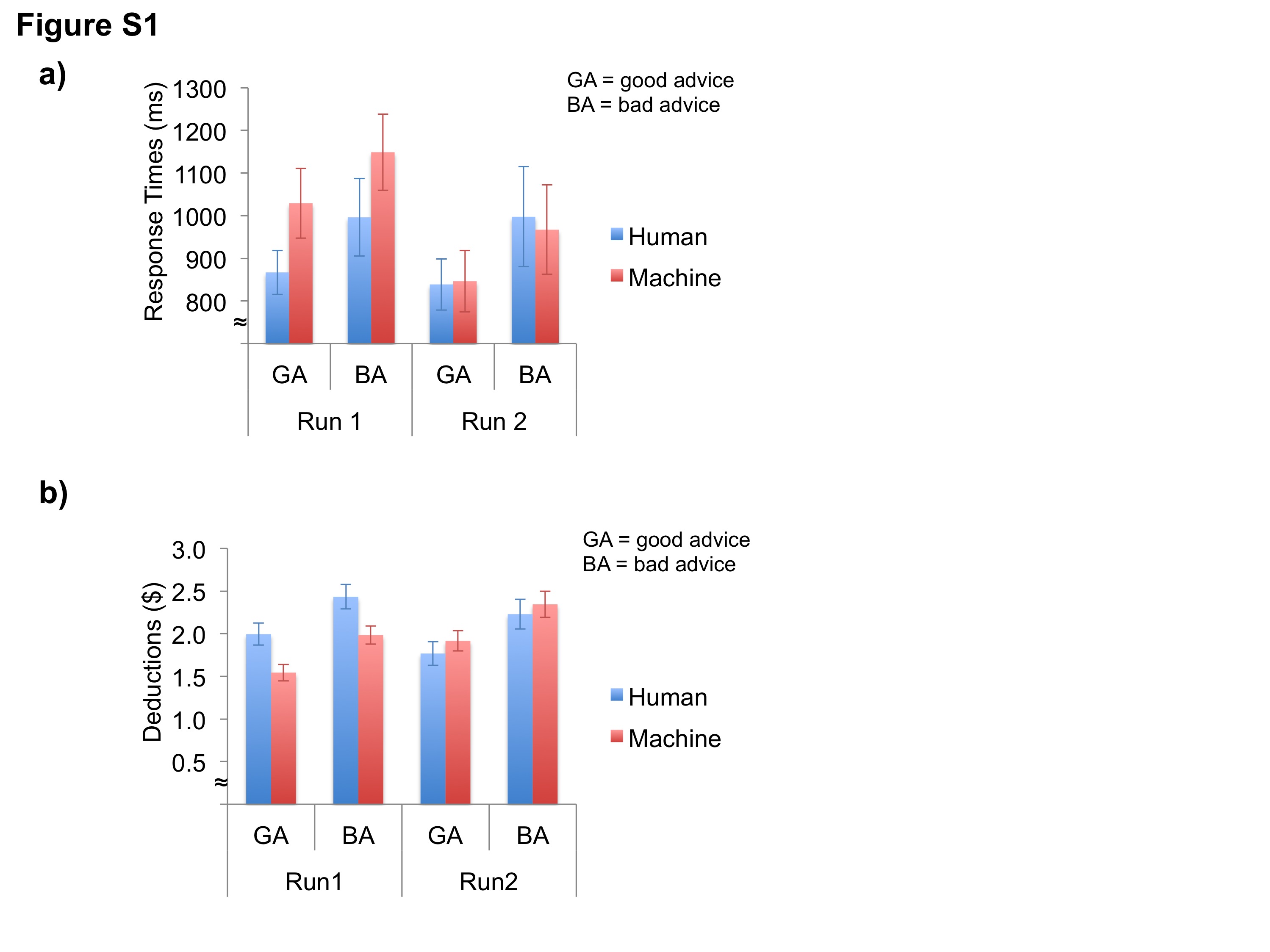

Supplement: Supplementary file 1 [file Image_1.JPEG]

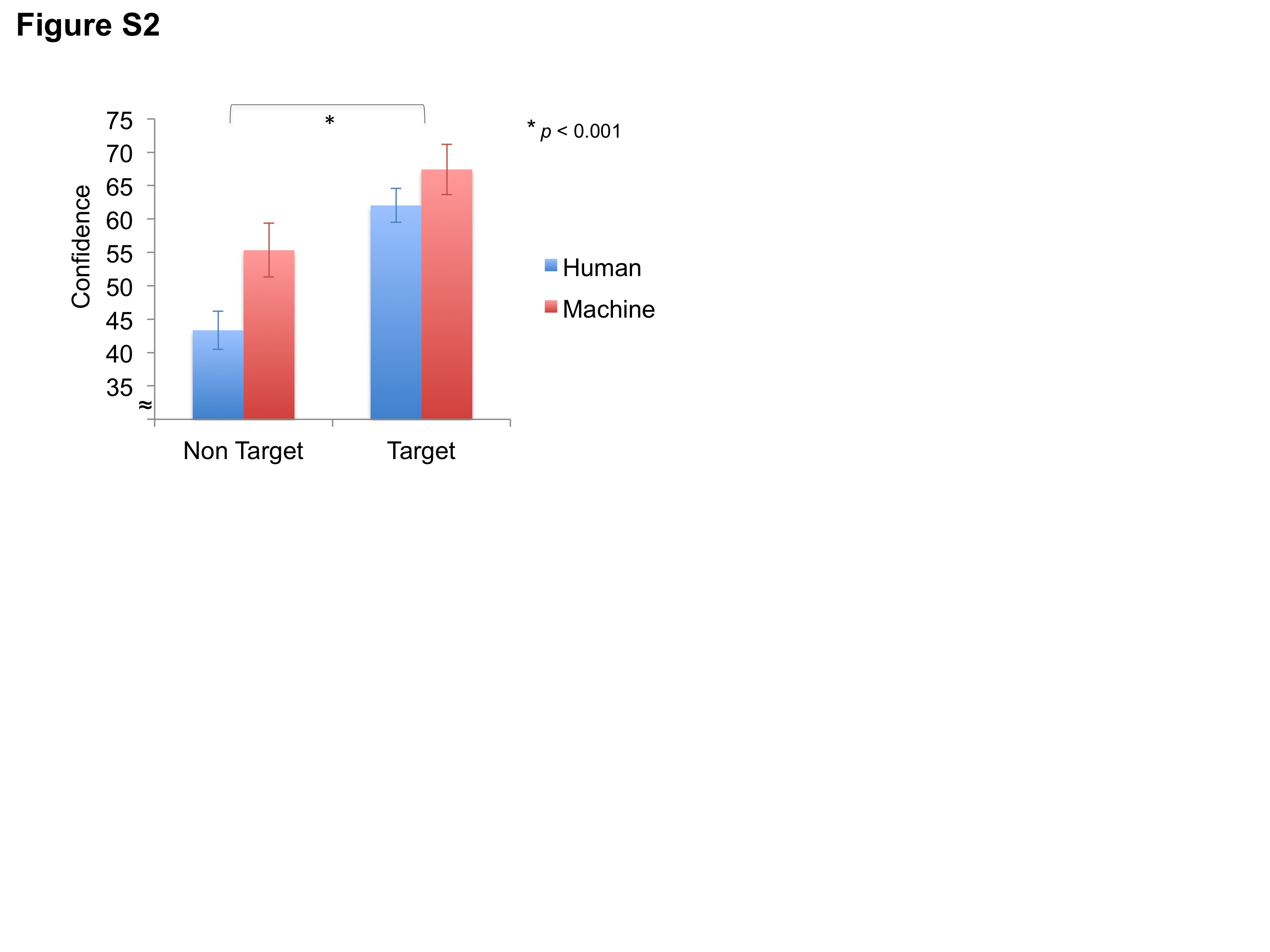

Supplement: Supplementary file 2 [file Image_2.JPEG]
