# Supplementary material for: Advice Taking from Humans and Machines: An fMRI and Effective Connectivity Study
Source: Front Hum Neurosci. 2016 Nov 4;10:542. doi: 10.3389/fnhum.2016.00542 (PMC5095979; doi:10.3389/fnhum.2016.00542)
Supplement: Supplementary file 3 [file Table_1.docx]

**Table S1**

**Human: Mr. Steve Williams**

Mr. Steve Williams (Human) is a trained luggage screener, with extensive knowledge in identifying illegal imports inside airline luggage. He has served the past 5 years in some of the busiest airports in the United States working at security checkpoints. He also specializes in antiterrorism and airport security and possesses extensive knowledge about the types of modern weapons and explosives commonly smuggled aboard aircraft. Mr. Williams has recently been appointed by the Transportation Security Administration (TSA) to oversee security operations at Dulles International Airport, which is one of the largest airports in the world.

**Machine: Automated Luggage Inspector**

The automated luggage inspector (Machine) is a diagnostic aid that has been programmed to identify hidden contraband in airline luggage. This Machine is based upon the technology traditionally used at major airport security checkpoints over the past 5 years. Its algorithms are sophisticated and are based on judgments using sensors different from those of the human visual system and can detect modern weapons and explosives smuggled aboard aircrafts. The automated luggage detector has recently been employed by the Transportation Security Administration (TSA) to enhance security operations at Dulles International Airport, which is one of the largest airports in the world.
