# Supplementary material for: Advice Taking from Humans and Machines: An fMRI and Effective Connectivity Study
Source: Front Hum Neurosci. 2016 Nov 4;10:542. doi: 10.3389/fnhum.2016.00542 (PMC5095979; doi:10.3389/fnhum.2016.00542)
Supplement: Supplementary file 4 [file Table_2.docx]

| **Table S2** |  |  |  |
| --- | --- | --- | --- |
| **Category** | **Human** | **Machine** | **Statistics** |
|  | **Demographics df = 22** | | |
| **Age** | 20.33 ± 2.55 | 20.42 ± 2.75 | *t* = -0.08, *p* = .939 |
| **Education** | 14.08 ± 2.35 | 14.13 ± 1.65 | *t* = -0.50, *p* = .960 |
| **Handedness** | 96.53 ± 8.31 | 92.49 ± 6.77 | *t* = 1.31, *p* = .205 |
| **Gender (male/female)** | 7/5 | 6/6 | *χ*^2^ = 0.17, *p* = .683 |
|  | **Complacency-Potential Rating Scale (CPS)** | | |
| **Confidence** | 15.17 ± 2.13 | 14.50 ± 1.78 | *t* = 0.83, *p* = .414 |
| **Reliance** | 9.50 ± 1.68 | 10.33 ± 1.78 | *t* = -1.18, *p* = .250 |
| **Trust** | 8.58 ± 2.28 | 8.92 ± 1.44 | *t* = -0.43, *p* = .672 |
| **Safety** | 6.25 ± 1.71 | 6.75 ± 2.09 | *t* = -0.64, *p* = .529 |
|  | **Interpersonal Reactivity Index (IRI)** | | |
| **Perspective Taking** | 28.25 ± 2.30 | 28.33 ± 3.37 | *t* = -0.71, *p* = .944 |
| **Fantasy Scale** | 19.33 ± 2.84 | 20.25 ± 2.80 | *t* = -0.80, *p* = .434 |
| **Empathic Concern** | 21.67 ± 5.07 | 22.33 ± 2.39 | *t* = -0.41, *p* = .684 |
| **Personal Distress** | 20.75 ± 2.80 | 20.67 ± 2.96 | *t* = 0.71, *p* = .944 |
|  | **NEO Five-Factor Inventory (NEO-FFI)** | | |
| **Neuroticism** | 31.33 ± 4.89 | 32.67 ± 3.94 | *t* = -0.74, *p* = .470 |
| **Extraversion** | 41.92 ± 3.37 | 40.42 ± 3.26 | *t* = 1.11, *p* = .280 |
| **Openness** | 37.75 ± 3.60 | 36.92 ± 4.72 | *t* = 0.49, *p* = .631 |
| **Agreeableness** | 38.67 ± 4.05 | 41.00 ± 4.51 | *t* = -1.33, *p* = .196 |
| **Conscientiousness** | 41.50 ± 3.56 | 42.17 ± 3.49 | *t* = -0.46, *p* = .647 |
|  | **National Technology Readiness Survey (NTRS)** | | |
| **Optimism** | 37.58 ± 4.87 | 39.08 ± 4.54 | *t* = -0.78, *p* = .444 |
| **Innovativeness** | 21.75 ± 4.20 | 24.83 ± 4.24 | *t* = -1.79, *p* = .087 |
| **Discomfort** | 31.00 ± 5.21 | 31.50 ± 5.02 | *t* = -0.24, *p* = .813 |
| **Insecurity** | 30.33 ± 5.68 | 29.08 ± 3.85 | *t* = 0.63, *p* = .534 |
|  | **Propensity to Trust (PTT)** | | |
| **Trust towards Automation** | 19.83 ± 2.21 | 20.42 ± 2.07 | *t* = -0.67, *p* = .511 |
|  |  |  |  |
