# Supplementary material for: Advice Taking from Humans and Machines: An fMRI and Effective Connectivity Study
Source: Front Hum Neurosci. 2016 Nov 4;10:542. doi: 10.3389/fnhum.2016.00542 (PMC5095979; doi:10.3389/fnhum.2016.00542)
Supplement: Supplementary file 5 [file Table_3.docx]

| **Table S3** |  |  |  |  |  |
| --- | --- | --- | --- | --- | --- |
|  | *F* (1,22) value | Cluster Size (mm^3^ ) | x | y | z |
| **Decision Phase** | | | | | |
| *Advice* |  |  |  |  |  |
| Right orbitofrontal cortex | 13.14 | 673 | 18 | 45 | -18 |
|  |  |  |  |  |  |
| **Feedback Phase** | | | | | |
| *Advice* |  |  |  |  |  |
| Right middle frontal gyrus | 16.47 | 4848 | 36 | 18 | 57 |
| Right superior parietal lobule | 13.05 | 2010 | 21 | -45 | 57 |
| Right putamen | 12.18 | 1867 | 33 | -3 | 3 |
| Right posterior cingulate cortex | 12.47 | 4937 | 6 | -51 | 15 |
| Right head of the caudate | 14.27 | 1968 | 9 | 12 | -9 |
| Left orbitofrontal cortex | 12.30 | 3348 | -9 | 48 | -15 |
| Left precentral gyrus | 15.29 | 4486 | -24 | -24 | 63 |
| Left subcallosal gyrus | 13.08 | 2204 | -12 | 3 | -12 |
| Left middle frontal gyrus | 12.05 | 2553 | -33 | 25 | 60 |
| Left dorsolateral prefrontal cortex | 15.05 | 2228 | -42 | 36 | 12 |
| Left inferior frontal gyrus | 15.75 | 1778 | -36 | 27 | -6 |
|  |  |  |  |  |  |
