# Supplementary material for: Advice Taking from Humans and Machines: An fMRI and Effective Connectivity Study
Source: Front Hum Neurosci. 2016 Nov 4;10:542. doi: 10.3389/fnhum.2016.00542 (PMC5095979; doi:10.3389/fnhum.2016.00542)
Supplement: Supplementary file 6 [file Table_4.docx]

| **Table S4** |  |  |  |  |  |
| --- | --- | --- | --- | --- | --- |
|  | *t* (23) value | Cluster Size (mm^3^) | x | y | z |
| **Decision Phase** | | | | | |
| *Decision* |  |  |  |  |  |
| Right superior temporal gyrus | 4.57 | 156 | 51 | -15 | 12 |
| Right inferior parietal lobule | 4.94 | 652 | 54 | -33 | 24 |
| Right postcentral gyrus | 4.62 | 259 | 51 | -12 | 54 |
| Right precentral gyrus | 6.08 | 52 | 21 | -24 | 72 |
| Right lingual gyrus | -5.04 | 348 | 9 | -84 | -12 |
| Left cingulate gyrus | 5.10 | 346 | -12 | -15 | 42 |
| Left postcentral gyrus | 5.26 | 310 | -9 | -45 | 63 |
| Left superior temporal gyrus | 4.23 | 350 | -63 | -3 | 0 |
|  |  |  |  |  |  |
