# Supplementary material for: Advice Taking from Humans and Machines: An fMRI and Effective Connectivity Study
Source: Front Hum Neurosci. 2016 Nov 4;10:542. doi: 10.3389/fnhum.2016.00542 (PMC5095979; doi:10.3389/fnhum.2016.00542)
Supplement: Supplementary file 7 [file Table_5.docx]

| **Table S5** |  |  |  |  |  |
| --- | --- | --- | --- | --- | --- |
|  | *t* (23) value | Cluster Size (mm^3^ ) | x | y | z |
| **Feedback Phase** | | | | | |
| *Performance* |  |  |  |  |  |
| Right inferior parietal lobule | 5.21 | 896 | 48 | -36 | 30 |
| Right frontal eye fields | 4.91 | 574 | 48 | 21 | 45 |
| Right middle occiptial gyrus | 5.01 | 429 | 36 | -90 | 9 |
| Right putamen | 4.43 | 703 | 30 | -6 | 6 |
| Right cingulate gyrus | 4.68 | 585 | 24 | -45 | 24 |
| Left frontal eye fields | 4.99 | 1221 | -12 | 48 | 51 |
| Left dorsolateral prefrontal cortex | 5.32 | 598 | -48 | 42 | 18 |
| Left inferior occipital gyrus | 4.57 | 1155 | -39 | -93 | -15 |
| Left angular gyrus | 4.85 | 1198 | -54 | -66 | 42 |
|  |  |  |  |  |  |
